# Inhibition of the Nuclear Factor-κB Pathway Prevents Beta Cell Failure and Diet Induced Diabetes in *Psammomys obesus*

**DOI:** 10.1371/journal.pone.0013341

**Published:** 2010-10-11

**Authors:** Josefine Friberg, Morten F. Tonnesen, Schott Heller, Flemming Pociot, Thóra B. Bödvarsdottir, Allan E. Karlsen

**Affiliations:** 1 Diabetes Inflammation, Hagedorn Research Institute, Gentofte, Denmark; 2 Beta-cell Regeneration, Hagedorn Research Institute, Gentofte, Denmark; 3 Hagedorn Research Institute, Gentofte, Denmark; 4 Department of Clinical Sciences, University Hospital MAS, Lund University/CRC, Malmö, Sweden; 5 Diabetes and Obesity Pharmacology Novo Nordisk A/S, Maaloev, Denmark; University of Bremen, Germany

## Abstract

**Background:**

High doses of anti-inflammatory drugs, such as aspirin and salicylates, improve glucose metabolism in insulin resistant and type 2 diabetic patients. It has also been shown that the glucose lowering effect is related to the unspecific ability of these drugs to inhibit inhibitor kinaseβ (IKKβ). In this study we have investigated the effect of a selective IKKβ-inhibitor on beta cell survival and the prevention of diet induced type 2 diabetes in the gerbil *Psammomys obesus (P. obesus)*.

**Methodology/Principal Findings:**

*P. obesus* were fed a diabetes inducing high energy diet for one month in the absence or presence of the IKKβ-inhibitor. Body mass, blood glucose, HbA_1C_, insulin production and pancreatic insulin stores were measured. The effects on beta cell survival were also studied in INS-1 cells and primary islets. The cells were exposed to IL-1β and subsequently reactive oxygen species, insulin release and cell death were measured in the absence or presence of the IKKβ-inhibitor. In primary islets and beta cells, IL-1β induced the production of reactive oxygen species, reduced insulin production and increased beta cell death, which were all reversed by pre-treatment with the IKKβ-inhibitor. In *P. obesus* the IKKβ-inhibitor prevented the development of hyperglycaemia and hyperinsulinaemia, and maintained pancreatic insulin stores with no effect on body weight.

**Conclusions/Significance:**

Inhibition of IKKβ activity prevents diet-induced diabetes in *P. obesus* and inhibits IL-1β induced reactive oxygen species, loss of insulin production and beta cell death *in vitro*.

## Introduction

Obesity is characterised by a state of low-grade inflammation [1]–[3], which may contribute to the development of insulin resistance and type 2 diabetes [3], [4]. Type 2 diabetes is characterized by insulin resistance, pancreatic beta cell dysfunction and reduced beta cell mass [5]. Although interventions that reduce inflammatory parameters have been associated with improved insulin and glucose metabolism during insulin resistance and type 2 diabetes [2], [6], the mechanisms connecting inflammation and diabetes are still unclear.

Inflammatory processes are characterised by the migration of proliferating white blood cells from the circulation to the tissues and activated macrophages and cytokines released from the adipose tissue have been associated with insulin resistance [7], [8]. Whereas TNFα has been associated with peripheral insulin resistance [3], islet inflammation and increased beta cell death have in particular been associated with the cytokine IL-1β [9], [10]. IL-1β may be produced by beta cells themselves or by homing macrophages within the pancreas. The involvement and therapeutic potential of IL-1β in the treatment of type 2 diabetes was recently demonstrated by treatment with the IL-1β receptor antagonist Anakinra, which resulted in improved beta cell secretory function and glycaemia control in human type 2 diabetic patients [11].

The transcription factor nuclear factor-kappa-B (NFκB) is central in the regulation of inflammatory responses and control of the innate immune system [12], [13]. Both TNF-α and IL-1β stimulate NFκB activation. NFκB is activated by a protein complex, where interaction between the inhibitor kinase (IKK) proteins -α, -β, -γ leads to phosphorylation of the inhibitor κB (IκB) protein. IκB is subsequently ubiquitinated and targeted for degradation by the proteasome, allowing release and translocation of NFκB to the nucleus and subsequent gene transcription [14], [15].

In animal models, the lack of IKKβ in immune cells protects the animals from insulin resistance during High fat feeding [16]. It has also been shown that the anti-inflammatory drug salicylate improves insulin resistance in diabetic rodents as well as glycaemia in obese and diabetic patients, at least in part, through the inhibition of the NFκB pathway [17], [18].

In most cell-types regulation of inflammatory pathways is associated with the induction of oxygen-derived free radicals, often referred to as reactive oxygen species (ROS). Increased ROS production has been demonstrated to be a unifying mechanism in endothelial cell dysfunction and late diabetic complications [19]. Additionally, ROS seems to be associated with insulin resistance in adipocytes, as normalisation of ROS levels reverses the inflammatory response and increases insulin sensitivity of these cells [20]. Observations in beta cells have shown that IL-1β mediated toxicity is in part mediated through induced production of reactive oxygen species in particular nitric oxide (NO) [21], [22].

We have utilized the gerbil *Psammomys obesus* (*P. obesus*) in our studies, as it is a rodent model of nutritionally induced type 2 diabetes. When transferred from their normal low-calorie saltbush diet to a more energy rich diet (regular rodent laboratory chow) the majority of the animals develop diabetes within a few weeks [23], [24]. This animal model is characterized by a fast depletion of insulin stores as a diabetic phenotype develops [24]. Similar to humans, these animals display increased pancreatic inflammation and associated beta cell death [9], [25].

The aim of the present study was to evaluate if inhibition of the IKKβ and NFκB pathways could prevent 1) IL-1β mediated beta cell toxicity *in vitro* and 2) diet induced diabetes *in vivo*. This was done using a selective IKKβ-inhibitor originally developed against steroid-resistance asthma [26].

## Results

### Inhibition of the NFκB pathway inhibits IL-1β induced beta cell failure in INS-1 cells

**Figure 1:**
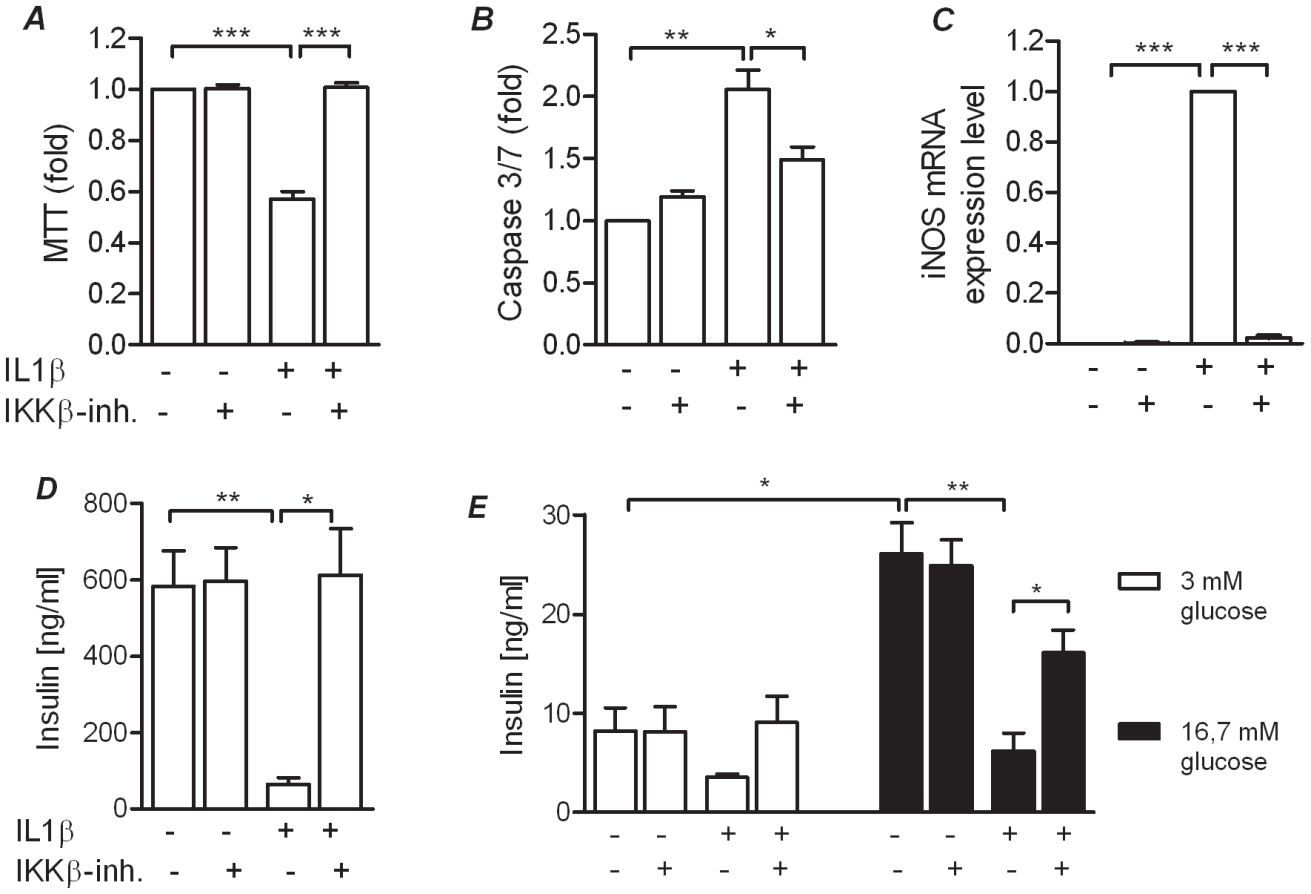
Prevention of IL-1 mediated beta cell toxicity in INS-1 cells by an IKKβ-inhibitor. Rat insulinoma (INS-1) cells were incubated in the presence and absence of IL-1β and the IKKβ-inhibitor for 48 hours. *A*: mitochoiondrial activity, a measurement of viability was determined by the MTT assay (Promega, USA). *B*: Cell death was measure as caspase 3/7 activity. *C*: iNOS mRNA expression was quantified using RT-PCR after cells had been incubation for 24 hours in the presence and absence of IL-1β and the IKKβ-inhibitor. *D*: Accumulated Insulin in the media was measured after 48 hours incubation in the presence or absence of IL-1β and the IKKβ-inhibitor. *E*: GSIS was performed after 48 hours incubation in the presence or absence of IL-1β and the IKKβ-inhibitor. All data are shown as the mean ± S.E.M. (*p<0,05; **p<0,01; ***p<0,001).

We first tested our hypothesis that inhibition of the NFκB pathway with the IKKβ-inhibitor would rescue beta cells against IL-1β-induced toxicity. This was done in INS-1 cells by measuring viability and apoptosis during IL-1β exposure. Forty-eight hours of IL-1β exposure reduced mitochondrial activity around two-fold (fig. 1*A*) in parallel with an increased caspase 3/7 activity (fig. 1*B*). Co-incubation with the IKKβ-inhibitor completely restored mitochondria activity and significantly reduced caspase 3/7 activity.

Since previous observations in beta cells have shown that IL-1β mediated beta cell destruction is in part mediated through nitric oxide (NO) production [27], we also measured expression levels of inducible nitric oxide synthase (iNOS). As expected iNOS transcription was massively induced in response to 24 hours IL-1β exposure, which was fully prevented by co-incubation with the IKKβ-inhibitor (Fig. 1*C*).

To examine if the IKKβ-inhibitor also protected against IL-1β induced loss of insulin secretion, accumulated and GSIS were measured. This was done after 48 hours incubation in the absence or presence of IL-1β and with or without the IKKβ-inhibitor. As seen from fig. 1*D* accumulated insulin production was halted by IL-1β exposure, but completely unaffected when the IKKβ-inhibitor was also present. Analysis of GSIS in control cells demonstrated a more than three-fold induction (8,2 vs. 26,1 ng/ml insulin) (Panel *E*). Cells exposed to IL-1β for 2 days displayed a diminished insulin production at both basal glucose (Panel *E white columns*, although non-significant) and followed stimulation at high glucose (Panel *E. black columns*). Co-incubation with the IKKβ-inhibitor significantly protected the cells against the IL-1β-induced decrease in insulin secretion (16,1 vs. 6,2 ng/ml insulin).

### IL-1β induced radical formation prevented by the IKKβ-inhibitor

**Figure 2:**
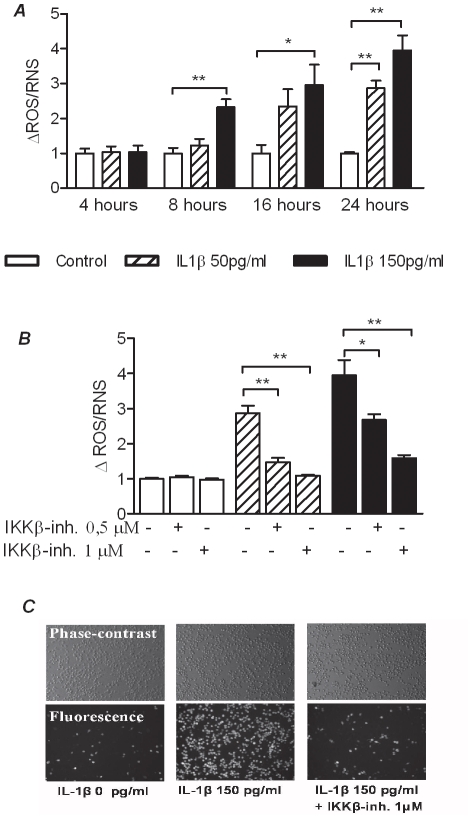
Prevention of IL-1 mediated ROS in INS-1 cells by an IKKβ-inhibitor. *A*: ROS was measured in Krebs-Ringer bicarbonate buffer with 10 µM fluorescent probe CM-H2DCFDA (Molecular probes) following incubation of the cells with and without IL-1β for 4, 8, 16 and 24 hours. Results are shown as delta fluorescence (t 90 min – t45 min). *B*: ROS was measured after 24 hours incubation in the presence or absence of IL-1β and the IKKβ-inhibitor. *C*: Microscopy of INS-1 after 24 hours incubation in the presence or absence of IL-1β and the IKKβ-inhibitor (magnification x10). Phase contrast (upper photos) and fluorescence (lower photos) after incubation with 10 µM fluorescent probe (CM-H_2_DCFDA). All data are shown as the mean ± S.E.M. (*p<0,05; **p<0,01; ***p<0,001).

To further characterize the effects of IL-1β on INS-1 cells, the production of radical formation was measured by H_2_DCFDA. H_2_DCFDA is normally referred to as an indicator of ROS despite its affinity for both reactive oxygen and nitrogen species (Table 1) [28]. We confirmed and extended previous observations demonstrating that that IL-1β exposure caused the level of ROS to rise in a time and concentration dependent manner with maximal production after 24 hours (Fig. 2*A*). Co-incubation with the IKKβ-inhibitor reduced the ROS production in a dose-dependent manner (Fig. 2*B*). 0,5 µM of the IKKβ-inhibitor attenuated the effect of 50 pg/ml IL-1β whereas a double dose, 1 µM was required to inhibit the effect of the 150 pg/ml IL-1β treatment. A macroscopic examination revealed that not only a subpopulation of dividing cells, but all cells were affected by the treatments (Fig. 2*C*).

**Table 1:** Fluorescence response of H_2_DCFDA to various reactive oxygen and reactive nitrogen species [28].

Reactive oxygen ornitrogen species	H_2_DCF affinity (%)
Hydroxyl radical (HO·)	100
Peroxynitrite anion (ONOO^–^)	89
Peroxyl radical (ROO·)	10
Hydrogen peroxide (H_2_O_2_)	3
Nitric oxide (NO)	2
Hypochlorite anion (^–^OCl)	1
Superoxide anion (·O_2_^–^)	1
Singlet oxygen (1O2)	0,4

### Effect of IL-1β and the IKKβ-inhibitor using primary newborn rat islets

**Figure 3:**
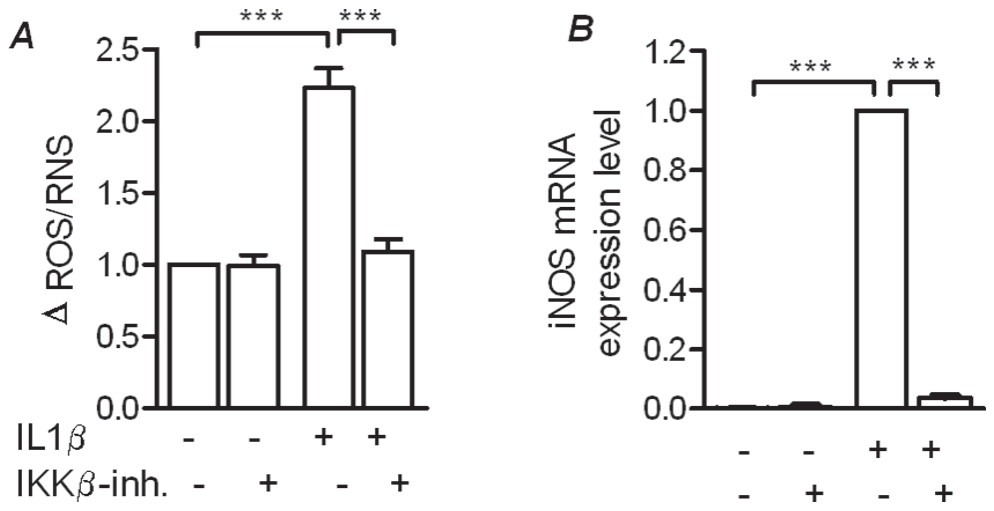
Prevention of IL-1β mediated ROS in newborn rat islets of Langerhans by an IKKβ-inhibitor correlates with transcription of iNOS. *A*: In newborn rat islets ROS was measured after 24 hours incubation in the presence or absence of IL-1β and the IKKβ-inhibitor. *B*: iNOS mRNA expression was quantified using RT-PCR after the islets had been incubation for 24 hours in the presence and absence of IL-1β and the IKKβ-inhibitor. All data are shown as the mean ± S.E.M. (*p<0,05; **p<0,01; ***p<0,001).

To evaluate if the effects observed in the INS-1 beta cell line could be observed in primary beta cells, we isolated newborn rat islets and exposed them to IL-1β in the absence or presence of the IKKβ-inhibitor. We found that IL-1 β increased the production of ROS by more than two-fold and this effect was attenuated by by the IKKβ-inhibitor (fig. 3*A*). In parallel, iNOS expression was significantly up-regulated in IL-1β, which was prevented by the NFκB pathway inhibitor (Fig. 3*B*).

### Effects of IL-1β and IKKβ-inhibition on islets of Langerhans isolated from *P. obesus*

**Figure 4:**
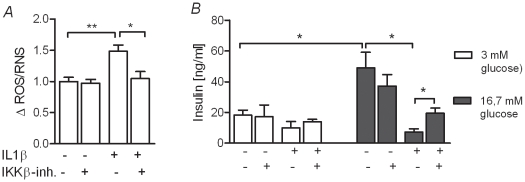
Islets from *P. obesus* are rescued from of IL-1β induced ROS production and diminished insulin production by an IKKβ-inhibitor. *A*: Islets from healthy adult *P. obesus* were incubated in the presence and absence of L-1β and the IKKβ-inhibitor for 24 hours before ROS was measured. *B*: GSIS was performed after 6 days incubation in the present or absent of IL-1β and the IKKβ-inhibitor. All data are shown as the mean ± S.E.M. (*p<0,05; **p<0,01; ***p<0,001).

To evaluate the *in vivo* potential of the IKKβ-inhibitor in the prevention of type 2 diabetes, the animal model *P. obesus* was selected. First we established the effects of the mouse IL-1β and the IKKβ-inhibitor on islets isolated from healthy adult animals. We measured the production of ROS and insulin following IL-1β exposure with or without co-incubation with the IKKβ-inhibitor. Exposure to IL-1β for 24 hours increased the production of ROS, which was attenuated by the inhibitor (fig. 4*A*). At basal glucose insulin secretion was not different between control islets and islets exposed to IL-1β or the IKKβ-inhibitor (fig. 4*B* *white columns*). However, at high glucose (fig. 4*B* *black columns*) IL-1β significantly reduced insulin secretion (from 49,0 to 7,2 ng/ml), an effect which was significantly attenuated in the presence of the IKKβ-inhibitor (19,5 vs. 7,2 ng/ml insulin).

### IKKβ-inhibition prevents diet induced diabetes in *P. obesus*

**Figure 5:**
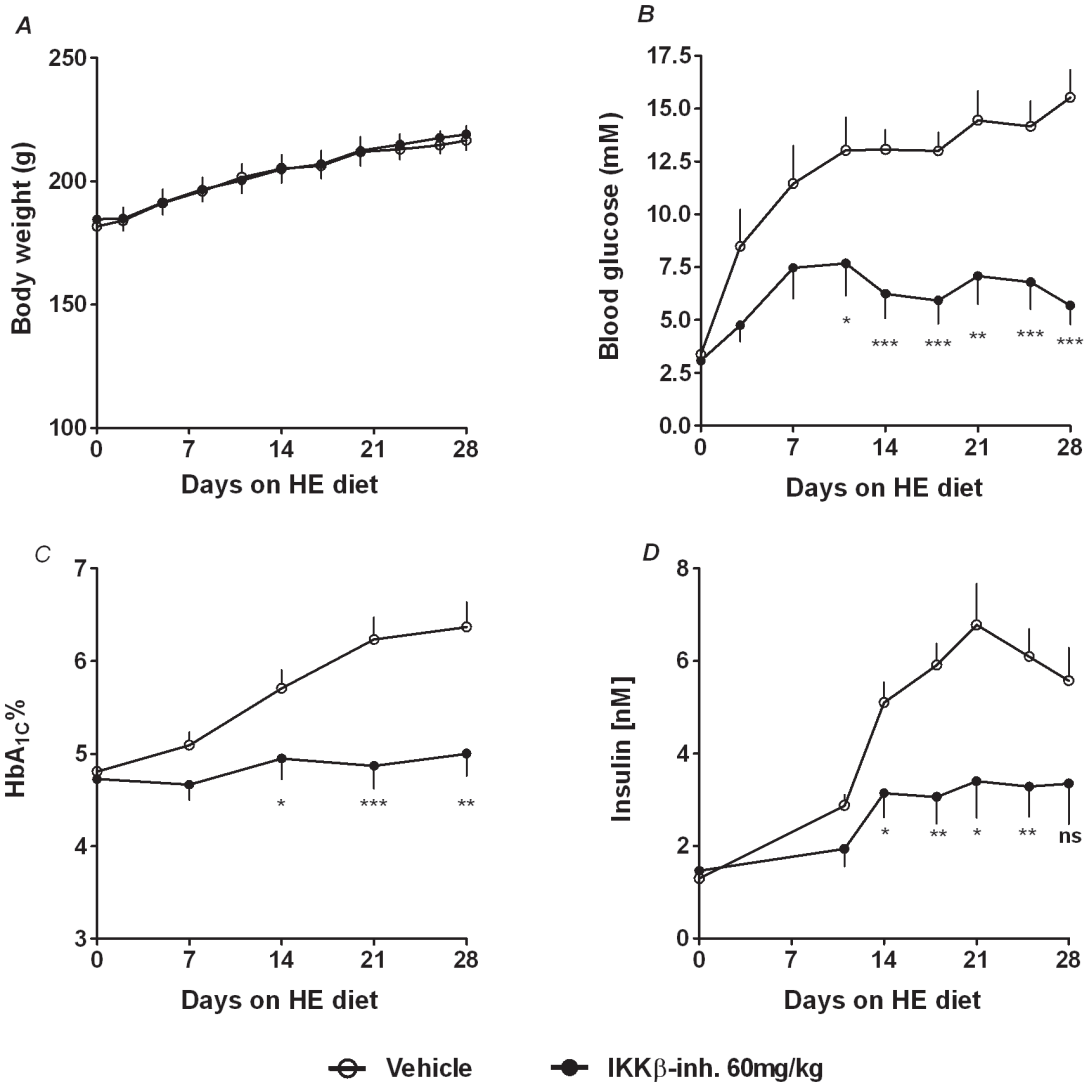
IKKβ inhibition prevents diet induced diabetes in *P. obesus*. Diabetes-prone male and female *P. obesus*, age of 13–14 weeks, were transferred from *ad libitum* low energy diet to *ad libitum* high energy diet. At the same day (day 0) treatment was initiated with vehicle (open circles n = 12) or with the IKKβ-inhibitor (black circles n = 10). *A*: Body weight was measured daily. *B*: Non fasting blood glucose was measured twice weekly. *C*: HbA1c was measured once weekly. *D*: Insulin was measured at day 0, 11 and then twice a week through the rest of the study. All data are shown as the mean ± S.E.M. (*p<0,05; **p<0,01; ***p<0,001).

We next examined if inhibition of the NFκB pathway could prevent diet induced diabetes. We used the diabetes prone *P. obesus* on a high energy diet treated with vehicle or 60 mg/kg/day IKKβ-inhibitor for 28 days. As seen in fig. 5*A* the inhibitor treatment had no effect on body weight gain. Vehicle treated animals developed diet induced diabetes during the 28 days on a high-energy diet (fig. 5*B*). Within one week the animals had a significant increase in non-fasting BG levels (from 3,4 mM at day one to above 10 mM at day 7 (p<0,001)), which was further increased to more than 15 mM by day 28. Hyperglycaemia was accompanied by a significant increase in HbA_1_C (fig. 5C), with an average of 4,8±0,1% at day zero compared to 6,4±0,3% at day 28 (p<0,001). Vehicle treated animals significantly increased plasma insulin levels until day 21 where hyperinsulinaemia was declining (fig. 5D) (day 1: 1,3±0,1 nM vs. day 21: 6,8±0,9 nM; p<0,001; day 28: 5,6±0,7 nM). Animals treated with the IKKβ-inhibitor showed no signs of diet induced diabetes during the 28 days on a high-energy diet. The levels of HbA_1_C (fig. 5C), BG (fig. 5B), and plasma insulin (fig, 5D) were significantly lower than the vehicle treated control animals and did not show a significant increase from day 0 to day 28.

**Figure 6:**
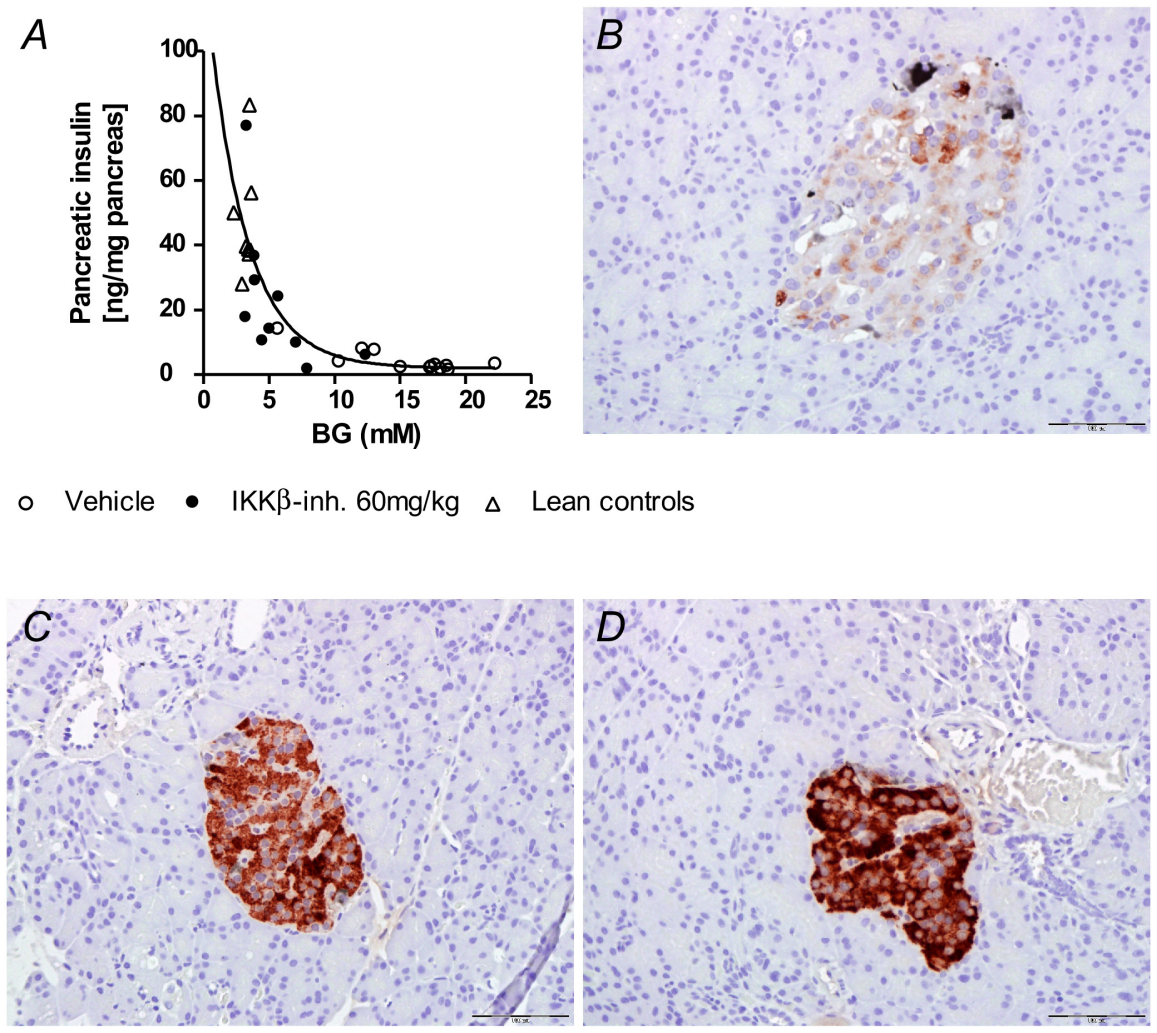
Effect of an IKKβ-inhibitor on pancreatic insulin content in *P. obesus*. Diabetes-prone *P. obesus*, age of 13–14 weeks, were transferred from *ad libitum* low energy diet to *ad libitum* high energy diet. At the same day (day 0) treatment was initiated with vehicle or with the IKKβ-inhibitor. A) Data shows blood glucose vs. pancreatic insulin content after 28 days of treatment with vehicle (open circles n = 12) or with the IKKβ-inhibitor (black circles n = 10); and from age matched non-diabetic animals feed *ad libitum* low energy diet (lean control) (open triangles n = 8). Immunostaining in reddish brown for insulin in tissue sections of pancreata from animals after 28 days of treatment with vehicle (B) or with the IKKβ-inhibitor (C); and from a age matched non-diabetic lean control animal (D).

To further evaluate the effects of treatment on beta cell function and capacity, we stained for insulin and measured total insulin content in acid ethanol extracts of pancreas from animals treated for 28 days with vehicle or the IKKβ-inhibitor in addition to age matched non-diabetic animals. As seen in figure 6A we found an inverse correlation between pancreatic insulin content and blood glucose. Furthermore, the treatment had a significant effect on pancreatic insulin supply, as reflected by a more than three-fold increase of pancreatic insulin content in animals treated with the IKKβ-inhibitor compared to the vehicle treated animals (day 28: IKKβ-inh. 26±7,2 µg vs. vehicle 7±1,5 µg; p<0,05). After 28 days on high energy diet insulin stainings also showed that most beta cells barely expressed insulin (fig. 6B). However, animals treated with the IKKβ-inhibitor showed clear and dense insulin staining of most beta cells (fig. 6C), which were comparable with insulin staining of beta cells in non-diabetic lean controls animals (fig. 6D).

## Discussion

The transcription factor NFκB is a “master switch” in inflammatory processes. NFκB is found in almost all cell types and is involved in cellular responses to stimuli such as stress, cytokines, free radicals, ultraviolet irradiation, oxidized LDL, and bacterial or viral antigens. A connection between inflammation, islet function and type 2 diabetes has been found. Non-specific inhibitors of the NFκB pathway, including aspirin and aspirin-related drugs, have shown positive therapeutic effects in diabetic patients, indicating the importance of NFκB activation in the development of type 2 diabetes [2], [6], [17], [18], [29].

As beta cell dysfunction and death in type 2 diabetes has been associated with cytokine-induced radical formation [25], we incubated INS-1 cells, rat islets and *P. obesus* islets with IL-1β and measured free radical formation, beta cell function and beta cell death. In isolated beta cells and islets, IL-1β increased the production of free radicals significantly, which was reflected in a loss of insulin production and beta cell death. In beta cells, IL-1β-induced free radical formation was shown in a time and dose dependent manner, with maximum production after 24 hours. IL-1β induced beta cell death has been associated with increased activity of NFκB. One key NFκB-regulated gene that is associated with beta cell death, is iNOS. iNOS is primarily responsible for the production of NO radicals [21], [22], and is normally silent, however IL-1β treatment induces a significant induction of the iNOS mRNA expression. We found that IL-1β up-regulated the expression of iNOS by greater than 500-fold in both beta cells and islets. IL-1β induced iNOS transcription, radical formation, loss of insulin production and beta cell death were all reversed by the IKKβ-inhibitor, indicating a link between iNOS activity, radical formation, beta cell function and death. A tight redox balance is important for the beta cell since it is weakly protected against oxidative damage, as a result of low levels of the defense proteins superoxide dismutase and glutathione peroxidise [30], [31]. There are many reactive species, each with various harmful effects. One example is the superoxide radical (O_2_*-), which is produced continuously as a by-product of oxidative phosphorylation, but is not very reactive. Similar to superoxide, the NO radical reacts slowly, however, when the two are combined together they form the highly toxic peroxynitrite radical (ONOO^−^) [32]. The probe used in our study to quantify radical formation has a low affinity for both NO and superoxide compared to peroxynitrite, and since peroxynitrite effectively oxidizes proteins, lipids and DNA, it is likely that IL-1β induced radicals caused beta cell failure and ultimately beta cell death.

*P. obesus* is a well established model of diet induced type 2 diabetes. When these animals are switched from low caloric density diet to a high caloric density diet they rapidly develop obesity, hyperglycaemia and hyperinsulinaemia [24], [33]. In this study, a high-energy diet resulted in a rapid rise in insulin levels in the vehicle treated animals. However, the higher level of insulin was not sufficient to maintain normal blood glucose control as both BG and subsequently HbA_1C_ continued to rise. After three weeks of a high-energy diet there was a decrease in insulin levels and a further deterioration in BG control. Loss of insulin production is expected to coincide with the loss of insulin production and beta cell destruction reported during the late stage of diabetes development in this animal model [24], [25], [33]. In animals treated with the IKKβ-inhibitor, the high-calorie diet only resulted in a transient increase in BG. However, a small counter-regulatory increase in insulin production was sufficient to prevent an incipient hyperglycaemia. The protective effect against hyperglycaemia by the treatment with the IKKβ-inhibitor was also reflected in HbA_1C_ levels as these were unchanged through the study.

It is not resolved as to whether the inhibitor treatment resulted in a short-term effect on beta cell function as a result of a lower demand for insulin due to increased insulin sensitivity, or if it has a permanent effect on beta cell survival. However, during the 4 week study, the animals treated with the IKKβ-inhibitor showed no sign of insulin depletion or beta cell failure. An early sign of beta cell failure in *P. obesus* is decreased pancreatic insulin content, which leads to decreased insulin production and reduced plasma insulin levels [24], [33]. When we analyzed pancreatic insulin content we found an inverse relationship between pancreatic insulin stores and blood glucose. We also found that animals treated with the IKKβ-inhibitor had significantly more pancreatic insulin content, as demonstrated by extraction and immunohistochemistry.

The presented data demonstrates that the inhibition of the NFκB pathway *in vitro* protects against beta cell failure and *in vivo* protects against hyperglycaemia and decreased pancreatic insulin content. Still, the *in vivo* effect on beta cell function could be a secondary effect of increased insulin sensitivity which our experiments did not investigate. However, previous studies have demonstrated that inhibition of IKKβ activity protects against lowered insulin sensitivity. For instance, knock out of IKKβ in hepatocytes and myeloid cells have shown to protect high fat fed mice against insulin resistance in both muscle, liver and fat [16], [34]. Furthermore, heterozygous deletion of *IKKβ* also protects against the development of insulin resistance both during high-fat feeding and in obese *Lep^ob/ob^* mice [18]. Non-steroidal anti-inflammatory drugs such as aspirin and salicylate also have a glucose lowering effect in both rodents and humans [2], [18], [29]. These drugs are cyclooxygenase but also function as non-selective inhibitors of NFκB activity, which has been ascribed as the mechanism connected to increased insulin sensitivity [17].

In summary, we have shown that the development of diet-induced diabetes in *P. obesus* was prevented by treatment with a selective IKKβ-inhibitor. The IKKβ-inhibitor also prevented cytokine induced loss of insulin production and beta cell death in primary islets and beta cells, possibly by inhibition of ROS production. This was also reflected *in vivo* since treated animals had much larger insulin reserves compared to the exhausted pancreata in vehicle treated animals. In conclusion, these data support the use of anti-inflammatory drugs in the prevention of type 2 diabetes.

## Methods

### IKKβ-inhibitor

The IKKβ-inhibitor was synthesized at Novo Nordisk A/S. The structure and properties have been published by Ziegelbauer and colleague [26]. In all *in vitro* experiments a pure form of the IKKβ-inhibitor was used whereas an enantiomeric mixture of the compound was used in the *in vivo* assay. Ziegelbauer and colleague [26] referred to the pure and the enantiomeric mixture of the IKKβ-inhibitor as COMPOUND A and COMPOUND B, respectively.

### Cell culture

A rat insulinoma cell line, INS-1 cells (a gift from Prof. Claes Wollheim, Geneva [35]) was maintained in RPMI 1640 medium with 11 mM glucose supplemented with 10% fetal bovine serum, 100 U/ml penicillin, 100 µg/ml streptomycin and 50 µM β-mercaptoethanol (all from Gibco-BRL). For preparation of cell culture for MTT, Caspase and insulin measurement, 50.000 cells/well were seeded in 96-well poly-L-lysine coated (to insure adherence) culture dishes (Cyto-well 96F. Nunc, Roskilde, Denmark). Two days after plating, the cells were pre-incubated for one hour with the IKKβ-inhibitor (0,5 or 0,1 mM) before IL-1β (mouse IL-1β, Sigma) (50 or 150 pg/ml) were added. Following exposure for the period of time indicated in the figures, insulin secretion, viability (measured as MTT activity), caspase 3/7 activity and production of ROS were quantified. For RT-PCR, 1,5 mil cells/well were seeded in 6-well culture dishes (Nunc, Roskilde, Denmark). Two days after plating, the cells were pre-incubated for one hour with the IKKβ-inhibitor (0,1 mM) before IL-1β (150 pg/ml) was added. Cells were exposed for 24 hours before RNA was extracted.

### *In vitro* islet studies in isolated islets of Langerhans from newborn Wistar rats

Pancreata from 3- to 5-day-old Wistar rats (Taconic, Ry, Denmark) were collagenase-digested and the islets were isolated by Percoll gradient purification (GE Healthcare, Denmark) as previously described [36]. Islets were maintained in RPMI 1640 supplemented with 1% penicillin/streptomycin, 10% newborn calf serum, 11 mM glucose (Gibco-BRL) and incubated in a 5% CO_2_ humidified atmosphere at 37°C. For the measurement of ROS, about fifteen islets/well were cultured in 96-well poly-L-lysine coated dishes (Cyto-well 96F), whereas 1000 islets/well were cultured in 6-well dishes (Nunc, Roskilde, Denmark) for RT-PCR. One day after plating, islets were pre-incubated for one hour with the IKKβ-inhibitor (0,1 mM) before incubation with IL-1β (150 pg/ml) for another 24 hours.

### *In vitro* islet studies in *P. obesus*

Isolated islets from healthy adult *P. obesus* on LE diet (Harlan; Jerusalem, Israel) were handpicked, as described [37], and incubated in RPMi 1640 supplemented with 1% penicillin/streptomycin, 10% newborn calf serum and 5 mM glucose (Gibco-BRL). About fifteen islets/well were cultured in extracellular matrix coated 96-well plates (Biological Industries, Israel). After 5 hours, the islets were pre-incubated for one hour with the IKKβ-inhibitor (0,1 mM) followed IL-1β (2 ng/ml) (human IL-1β, Sigma) exposure for 48 hours for ROS measurement, or 6 days for Glucose-Stimulated Insulin Secretion (GSIS).

### Cell viability and cell death

In INS-1 cells, viability was assessed by 3-4,5dimethylthiazol-2,5diphenyl-tetrazolium bromide (MTT) assay (Promega) and cell death by caspase 3/7 activity (Apo-ONE® Homogeneous Caspase-3/7 Assay, Promega); both assays according to the manufacturers instructions.

### Insulin release

Before measurement of GSIS the culture media were collected and frozen for quantification of accumulated insulin secretion. To determine GSIS, the cells or islets were washed in Krebs-Ringer buffer (115 mM NaCl, 4,7 mM KCl, 2,6 mM CaCl_2_, 1,2 mM KH_2_PO_4_, 1,2 mM MgSO_4_, 5 mM NaHCO_3_, 10 mM HEPES, 0,2% BSA, pH 7.4) containing 3.7 mM glucose, and pre-incubated for half a hour in the same buffer. The buffer was replaced with a fresh buffer containing 3.7 mM glucose for one hour before the buffer were collected (basal) and replaced with a buffer containing 16.7 mM glucose for an additional one hour (glucose stimulated). Supernatants were collected and frozen for subsequent insulin determination; using a rat-specific luminescence oxygen channeling immuno assay.

### Measurement of intracellular ROS production

5-(and-6)-chloromethyl-2′,7′-dichloro-dihydrofluorescein diacetate (H_2_DCFDA) (Molecular probes) is a cell-permeable dye that within the cells is cleaved by esterases to H_2_DCF. By the action of different ROS (see Table 1) H_2_DCF is subsequently oxidized to its fluorescent form [28]. At the indicated times cells or islets were washed with DPBS (Gibco-BRL) and incubated in the dark with 10 µM fluorescent probe H_2_DCFDA in DPBS. Fluorescence was measured on a NovoStar Microplate Reader (Ramcon, Denmark). Results are shown as delta fluorescence (t90 min – t45 min).

### Measurement of gene expression by real-time PCR

Cells were lysed in RLT buffer and islets in Qiazol Lysis Reagent before RNA extraction according RNeasy protocol (Qiagen). cDNA was transcribed using iScript cDNA Synthesis Kit (Bio-Rad laboratories). Samples were assayed on an Mx3000 (Stratagene) using probs and primers designed by Probe Library (Roche Diagnostics). The expression level of the gene of interest was normalized to the housekeeping gene 36B4 and analyzed by the ΔΔCt method [38].

### Animals

Male and female *P. obesus*, aged 7–9 weeks, were obtained from Harlan (Israel). Animals were housed three per cage under controlled conditions with a 12 hours light-12 hours dark cycle, temperature of 25°C and fed low energy chow (LE-2.4 kcal/g; Teklad, Jerusalem, Israel) and water *ad libitum*. At 10-11 weeks of age animals were challenged with a high energy diet (HE-3.1kcal/g; Purina 5008). Animals that showed sign of hyperglycaemia (BG>10 mM) or no signs (BG<8 mM) during the test period of 10 days were classified as diabetes-prone animals or diabetic-resistant animals, respectively. All animals were returned to LE diet. At the age of 14–15 weeks all diabetes-prone animals were normoglycemic again and transferred to an *ad libitum* high energy diet. Animals were divided into two groups, treated orally with either 2 ml/kg of either vehicle (10% 2-hydroxypropyl beta-cyclodextrin in saline (HPCD/saline)) or the IKKβ inhibitor (60 mg/kg in HPCD/saline) every morning, for 4 weeks. During this period body-weight (BW) was measured daily and morning blood glucose (BG), HbA_1C_ and insulin was measured one or two times a week. The study was approved by the Animal Experiments Inspectorate, Ministry of Justice, Denmark (Approval number 2001/561-460).

### Blood analysis

Blood samples was taken from the tail tip capillary into 10 µl glass capillary tubes and immediately suspended in buffer (500 µl of Biosen analysis buffer) and analysed for glucose on the test day on a Biosen S_Line (EKF-diagnostic, GmbH). For HbA_1C_ measurement 5 µl was immediately suspended in 250 µl of Hitachi haemolysate buffer and analyzed on a Hitachi 912 (Roche Diagnostics). For insulin measurement blood samples were taken from the tail tip capillary into 80 µl capillary EDTA tubes and immediately centrifugation at 5000 rpm for 5 minutes (at 4°C). Plasma was collected and frozen until measurement of insulin using a rat-specific luminescence oxygen channelling immuno assay.

### Pancreatic insulin content

At day 28 animals were anaesthetized by isoflurane before the pancreas was removed as described above. Total pancreas was weighted and sampled using “the smooth fractionator” technique as earlier described [39]. Two thirds of the pancreas was homogenized on a tissue-lyzer (Qiagen) at 25 Hz for 2 min in acid-ethanol (30 mM HCl in 75% ethanol) using 1 µl/mg tissue. The samples were placed on a rocker at 4°C overnight before centrifuged at 20000×g at 4°C for one hour. Supernatants were collected and the insulin concentration was quantified as described above.

### Pancreatic beta cell staning for insulin

One third of the pancreata from *Psammomys obesus* (sampled as described above) were fixed in 4% paraformaldehyde, followed by paraffin embedding. Sections were deparaffinized and rehydrated. Endogenous peroxidase was blocked by submersion in 0.5% H_2_O_2_ for 20 minutes before washed in Tris-buffer saline (TBS) with 0,1% Tween 20 (TBS-T). The remaining steps were carried out in an autostainer (DAKO Corp., Carpinteria, California, USA). Sections were incubated 10 minutes with Avidin blocking solution (DAKO) followed incubation for 10 minutes with Biotin blocking solution (DAKO), with a washing procedure in TBS-T between each step. Sections were incubated for 15 minutes with 10% rabbit serum (X0902, DAKO) in TBS-T before stained for 60 minutes for insulin with guinea pig anti-insulin serum (A0564, DAKO) 1∶500 in 7% rabbit serum and 3% rat serum in TBS-T. Sections were washed with TBS-T before incubated for 30 minutes with peroxidise labelled rabbit anti-guinea pig (P0141, DAKO) 1∶100 in TBS-T. Peroxidise reactivity were developed for 12 minutes with Nova Red (Vector laboratories, Burlingame, California, USA). Sections were washed in tap water before counterstained for 15 seconds with haematoxylin, dehydrated and mounted with Pertex.

### Statistical analysis

Data were analyzed with an unpaired t-test assuming equal variances. If variances were found to be different between groups, Welsh's correction was applied. Results are represented as mean ± SEM. *p*-values less than 0.05 are considered significant and are indicated with *, while *p*-values of *p*<0.01 and *p*<0,001 are marked with ** and ***, respectively.
